# Supplementary material for: A Critical View of the Application of the APEX Software (Aqueous Photochemistry of Environmentally-Occurring Xenobiotics) to Predict Photoreaction Kinetics in Surface Freshwaters
Source: Molecules. 2019 Dec 18;25(1):9. doi: 10.3390/molecules25010009 (PMC7017383; doi:10.3390/molecules25010009)
Supplement: Supplementary file 1 [file molecules-25-00009-s001.zip › 3.2.4_gcc-4.4.0.zip!/3.2.4_gcc-4.4.0/3.2.4_gcc-4.4.0/doc/octave/html/interpreter/Table-of-Input-Conversions.html]

Table of Input Conversions - Untitled


Next: Numeric Input Conversions,
Previous: Input Conversion Syntax,
Up: C-Style I/O Functions


---

#### 14.2.13 Table of Input Conversions

Here is a table that summarizes the various conversion specifications:

%d: Matches an optionally signed integer written in decimal. See Numeric Input Conversions. %i: Matches an optionally signed integer in any of the formats that the C language defines for specifying an integer constant. See Numeric Input Conversions. %o: Matches an unsigned integer written in octal radix. See Numeric Input Conversions. %u: Matches an unsigned integer written in decimal radix. See Numeric Input Conversions. %x, %X: Matches an unsigned integer written in hexadecimal radix. See Numeric Input Conversions. %e, %f, %g, %E, %G: Matches an optionally signed floating-point number. See Numeric Input Conversions. %s: Matches a string containing only non-whitespace characters. See String Input Conversions. %c: Matches a string of one or more characters; the number of characters read is controlled by the maximum field width given for the conversion. See String Input Conversions. %%: This matches a literal % character in the input stream. No corresponding argument is used.

If the syntax of a conversion specification is invalid, the behavior is
undefined. If there aren't enough function arguments provided to supply
addresses for all the conversion specifications in the template strings
that perform assignments, or if the arguments are not of the correct
types, the behavior is also undefined. On the other hand, extra
arguments are simply ignored.
